# Flood- and Weather-Damaged Homes and Mental Health: An Analysis Using England’s Mental Health Survey

**DOI:** 10.3390/ijerph16183256

**Published:** 2019-09-05

**Authors:** Hilary Graham, Piran White, Jacqui Cotton, Sally McManus

**Affiliations:** 1 Department, University of York, York YO10 5DD, UK; 2 Department of Environment and Geography, University of York, Wentworth Way, York YO10 5NG, UK; 3 Environment Agency, Leeds LS11 9AT, UK; 4 National Centre for Social Research, London EC1V 0AX, UK

**Keywords:** climate change, environment, emergency planning, extreme weather events

## Abstract

There is increasing evidence that exposure to weather-related hazards like storms and floods adversely affects mental health. However, evidence of treated and untreated mental disorders based on diagnostic criteria for the general population is limited. We analysed the Adult Psychiatric Morbidity Survey, a large probability sample survey of adults in England (*n* = 7525), that provides the only national data on the prevalence of mental disorders assessed to diagnostic criteria. The most recent survey (2014–2015) asked participants if they had experienced damage to their home (due to wind, rain, snow or flood) in the six months prior to interview, a period that included months of unprecedented population exposure to flooding, particularly in Southern England. One in twenty (4.5%) reported living in a storm- or flood-damaged home in the previous six months. Social advantage (home ownership, higher household income) increased the odds of exposure to storm or flood damage. Exposure predicted having a common mental disorder over and above the effects of other known predictors of poor mental health. With climate change increasing the frequency and severity of storms and flooding, improving community resilience and disaster preparedness is a priority. Evidence on the mental health of exposed populations is key to building this capacity.

## 1. Introduction

Climate change is increasing population exposure to weather-related hazards, such as extreme precipitation events, storms and flooding [1,2]. In the UK, the government has identified flooding as posing particularly high risks to people and communities [3]. Alongside damage to homes and businesses, there is disruption to domestic utilities and transport links [4]. Exposure to extreme weather events such as flooding results in ‘psychological casualties’ [5], with significant impacts on mental health.

Capturing the mental health impacts of extreme weather events such as storms and floods presents many research challenges. Large representative surveys are required to compare the mental health of exposed and non-exposed populations in analyses with measures of other factors (for example, an individual’s financial circumstances) that may explain associations between storm or flood exposure and mental ill-health. A review of evidence on the mental health of flood-exposed populations found that the large majority (77%) of studies did not include a comparison group and noted a lack of attention to potential confounders like socioeconomic status [6]. Additionally, robust studies require detailed measures of people’s mental health, including treated and untreated mental health disorders, based on clinically validated diagnostic criteria. Only well-resourced health surveys are equipped to collect such information for large population surveys [7].

This study uses a large, national and representative mental health survey in England to investigate (i) the social profile of those with recent experience of storm- and flood-related damage to their home and (ii) whether the experience independently predicts common mental disorder.

## 2. Materials and Methods

The Adult Psychiatric Morbidity Survey (APMS) is funded by England’s Department of Health and Social Care, commissioned by NHS Digital (Leeds, England), and conducted by the National Centre for Social Research and Leicester University. It is the primary source of information on the mental health of people living in England [8]. The most recent APMS (*n* = 7525) ran from May 2014 to September 2015, with fieldwork evenly distributed across this period. For the first time, the survey included the following question: ‘Has your home been damaged by wind, rain, snow or flood in the last six months’; it, therefore, covered the period November 2013 to September 2015. This period included four months, from December 2013 to March 2014, of severe winter storms and extensive flooding in the UK [9,10].

The survey is based on a stratified multi-stage random sample of the general population aged ≥16 years in private households. It includes detailed mental health screening tools and assessments to measure treated and untreated mental disorder assessed to the diagnostic criteria set out in the tenth edition of the International Classification of Diseases [11]. The questionnaire is administered face-to-face in people’s own homes, with the most sensitive information collected by computer self-completion. The overall response rate for the survey (57%) is in line with other national surveys of similar scale [12]. The analysis used specially developed weighting variables to adjust for known patterns of non-response according to area- and individual-level characteristics; by applying a multiplier to each participant, the sample was rendered representative of the wider population. Ethical approval for APMS 2014 was obtained from the West London National Research Ethics Committee, approval reference number 14/LO/0411. Data use approval for the analysis was secured via NHS Digital. Further methodological information is available elsewhere [8].

Outcome measure: The outcome variable was the presence of common mental disorder (CMD), based on the CIS-R (Clinical Interview Schedule-Revised), which covers non-psychotic symptoms in the past week [13]. The CIS-R operationalises standardised diagnostic criteria from the 10th International Classification of Disease [14]; algorithms were applied to responses to assess for depression, phobias, generalized anxiety disorder, obsessive compulsive disorder (OCD), panic attacks and ‘CMD not otherwise specified’. CMD was defined as the presence of one or more of these six disorders. Our analysis also used variables related to other experiences of distress. Posttraumatic stress disorder (PTSD) was measured by screening positive on the self-completed PTSD Checklist civilian version (PCL-C); it indicates the presence of trauma-related symptoms in the past week and that clinical assessment for PTSD is warranted [15]. Respondents were also asked about suicidal thoughts, non-fatal suicide attempts and self-harm (without suicidal intent) in both the face-to-face and self-completion sections of the interview. Use of mental health treatment and service use was established via questions about prescribed mental health medication and psychological (talking) therapy around the time of the interview.

Exposure measure: The exposure measure is the experience of damage from wind, rain, snow or flood to the home in the six months prior to interview. This period included the winter floods of 2013–2014 which were ‘one of, if not the most, exceptional periods of winter rainfall in at least 248 years’ [16]. However, the APMS question did not allow us to distinguish people affected by external flooding from sea, rivers, or surface flow from those affected by escape of water inside the house (e.g., leaking boilers and pipes), so we refer hereafter to ‘storm- and flood-related damage’. Data provided by a leading UK insurance company with broad and representative geographical exposure through a multi-channel distribution route [17] indicate that, over the 2013–2015 period, weather-related damage accounted for 46% of all claims due to storms, flood, freeze or escape of water, and 62% of all claims costs resulted from these causes. Most weather-related claims were due to storm damage (91%), with 6% due to flood damage and 3% due to freezing. The relative contribution to overall costs of weather-related claims was 79% from storm damage, 19% from floods and 2% due to freezing. Thus, the relative cost of an average flood-related claim is over 3.5 times greater than a storm-related claim. It should be noted these insurance-based data are likely to underestimate the costs of weather-related events; compared to owner occupiers, those in rented accommodation are less likely to have contents insurance (which provides cover for the financial cost of damage to, or loss of, personal possessions located within the home) and those living in more deprived communities are less likely to insure adequately against flood damage [18,19].

Covariates: We included individual-level socio-demographic measures: sex, age group (in ten-year bands), broad ethnic groups (White British, White other, Black, Asian, and mixed or other), highest educational qualification and current employment status, together with measures of financial strain (being in serious debt and payment arrears; experience of a major financial crisis in the past six months). Health-related behaviours associated with poorer mental health were also included: harmful drinking (Alcohol Use Disorders Identification Test-AUDIT) [20], smoking and drug dependence [21]. General health was measured by self-reported health status (poor, fair, good, very good or excellent), an item from the 12-item Short Form Health Survey (SF12) [22].

At household-level, we included quintiled equivalised household income, housing tenure and household composition, together with measures of housing conditions that may be related to both storm- and flood-related damage and CMD (presence of mould in the home and whether the participant was able to keep the home warm enough in winter) [23]. Area-level measures included area deprivation (quintiles of ranked Index of Multiple Deprivation (IMD) scores), which measures relative levels of deprivation in small areas of England called ‘lower layer super output areas’ [24], and urban/rural location. For the later, we used the government’s standard Rural–Urban Definition for Small-Area Geographies (RUC) classification; the derivation is complex but primarily based on the population density of hectare cells and proximity of other settlements [25]. To protect anonymity, region of residence was not included in the dataset released for the study.

Statistical analysis: Analysis was carried out using Stata v14, weighted and adjusted for the complex survey design. Descriptive sample profiling is presented in Table 1 and in the Supplementary Materials. Logistic regression was used to calculate unadjusted odds and adjusted odds for living in a storm- or flood-damaged home (aim 1) and for CMD (aim 2).

To investigate the social patterning of storm and flood damage (aim 1), we examined bivariate (unadjusted) associations by sex, age group, socioeconomic circumstances (employment status, household income, housing tenure) and area characteristics. Age and other factors with significant bivariate associations with living in a weather-damaged home were then entered into a multiple regression model with area deprivation (Table 2, Model 1). The final model retained factors significant in Model 1, together with age (Table 2, Model 2).

To establish whether recent damage to the home from storms and floods independently predicted CMD (aim 2), we began with an overall profile of CMD risk, examining bivariate associations with CMD for storm and flood exposure and for sex, age group, employment status, household income, housing tenure and area deprivation. We also identified other factors that, in these bivariate analyses, predicted CMD. Our first multivariate model (Table 3, Model 1) included storm and flood-related exposure together with socio-demographic and socio-economic factors that were significant predictors of both CMD and storm- and flood-related damage in bivariate analysis. Storm- and flood-related exposure was then added to this model of CMD risk (Table 3, Model 2). This final model additionally controlled for factors that were significantly related to CMD in our bivariate analysis and were also known from previous research to be predictors of CMD [26,27,28,29]. This allowed the independent contribution of storm- and flood-related exposure to the presence of CMD to be estimated after controlling for other relevant factors.

## 3. Results

One person in twenty (4.5%) reported living in a storm- or flood-damaged home in the six months prior to interview. Damage resulting in the participant being either confined to, or having to leave, their home was very rare (0.1%); no further analysis of this exposure was, therefore, undertaken.

Those experiencing storm or flood damage to their home had poorer mental health (Table 1). Storm- and flood-related damage was significantly associated with CMD (*p* ˂ 0.01). Associations with suicidal ideation (*p* < 0.01) and ever having attempted suicide were also significant (*p* ˂ 0.01). Associations with other mental disorders, including post-traumatic stress disorder (PTSD; included in Table 1), and with mental health treatment and service use were not significant.

### 3.1. Social Patterning of Storm and Flood Damage to the Home (Aim 1)

In bivariate analyses (Table 2), the factors significantly associated with storm- and flood-related damage include individual factors (sex, educational level, employment status, financial strain, health status, harmful drinking, smoking and drug dependence) and household factors (housing tenure, household income, household type). See Supplementary Data for full results.

In the adjusted model, sex, housing tenure and household income were independently predictive of storm or flood damage to the home (Table 2, Model 2). Odds were higher for men (women: OR 0.73, 95%CI 0.56; 0.94), for owner-occupiers compared with private renters (OR for private renters 0.57, 95%CI 0.39; 0.84) and for those in the highest household income quintile (lowest quintile: OR 0.60, 95%CI 0.38; 0.94).

### 3.2. Exposure to Storm- and Flood-Damaged Home as a Predictor of CMD (Aim 2)

In unadjusted analyses, the experience of storm or flood damage to one’s home increased the odds of CMD (Table 3). The association remained in the adjusted model (Table 3, Model 1), which took account of socio-demographic (sex, age group), socio-economic (employment status, housing tenure, household income) and area-level (IMD) factors, including factors significantly related to both flood exposure and to CMD. Flood exposure remained predictive of CMD in the final model (Table 3, Model 2), which additionally controlled for health status and stress-related context. In Model 2, storm or flood exposure increased the odds of CMD by 50% (OR 1.5, 95%CI 1.08; 2.07). The established predictors of CMD remained significant: being female, being young (age 16–24), having debt arrears, being in poor general health and being a hazardous or harmful drinker.

## 4. Discussion

Climate change is increasing the occurrence of extreme weather events, including storminess (including wind) and the frequency and intensity of floods [3,30,31,32,33]. The number of properties in the UK exposed to at least a 1 in 75-year flood risk is predicted to increase by 41% under a 2 °C temperature rise and by 98% under a 4 °C temperature rise [30]. In response to these threats, policy agencies at local, national and global level are seeking to develop disaster preparedness strategies [34,35,36]. Evidence on the mental health of affected populations is recognized to be an important element of such strategies; studies of populations experiencing flooding have found stronger associations with mental than physical health [37,38,39,40,41,42,43], with rates of CMDs like depression and anxiety elevated among those whose homes are flooded [6,44]. However, much of the evidence derives from studies of flood-exposed areas and not from general population surveys [6]. In particular, there are few large-scale studies with clinically validated measures of mental health of exposed and non-exposed populations [35].

Our study helps to address this gap. It is based on England’s major mental health survey (APMS), a large population survey that collects information on people’s mental health using validated tools. The most recent survey was conducted in the year following an exceptional period of flooding. December 2013–March 2014 saw extensive flooding which, compared to other periods of flooding, tended to be of longer duration [10]. Over 4.2 million flood warnings were issued [10] and over 10,000 residential properties were flooded. For the first time in the APMS series of surveys, the 2014/15 survey asked participants if they had experienced damage to their home (due to wind, rain, snow, flood) in the six months prior to interview. Using this exposure measure together with the detailed social data collected in the APMS, we investigated the social profile of those who experienced storm- and flood-related damage to their home and whether experience of recent damage independently predicted common mental disorder. As a population survey, the APMS enabled us to compare the mental health of exposed and non-exposed adults in analyses that controlled for other factors that may elevate the risk of CMD (for example, social disadvantage).

We found, firstly, that advantaged groups (owner-occupiers and higher income households) were more likely to report having experienced storm- or flood-related damage to their homes. Information on the location of study participants was not provided in the APMS dataset released for our study. National-level evidence points to an association between deprivation and flood risk in England [45,46,47]; however, this overall pattern masks different social distributions of risk between tidal and fluvial flooding [48]. Deprivation is associated with greater risk of tidal flooding, an association shaped by the geographical patterning of deprivation; coastal areas, including seaside towns, are home to many of England’s most deprived communities [48,49,50]. Social gradients are flatter for fluvial flood risk and, where gradients are evident, more advantaged groups tend to be at greater risk [46,48]. Among the most advantaged fifth of the population (those in the two least-deprived deciles) at risk from river flooding, over half (104,000) live in the South East [46]. The 2013–2014 floods were regionally concentrated, with the largest number (39%) of flooded homes in the South East [10], England’s most populous and most affluent region with the highest rates of property ownership [44,51,52,53].

We found, secondly, that experiencing storm- or flood-related damage in the home increased the risk of having a CMD, over and above the established predictors of poor mental health. As this suggests, our study confirmed the importance of known risk factors for CMD; the predictors evident in our study were: being female, aged 16–24, living in a deprived neighbourhood, being in debt arrears, having poorer general health, and harmful or hazardous patterns of alcohol use. Our study identified storm and flood exposure in the home as an additional risk factor. Our measure did not include those, who while avoiding storm or flood damage to their own home, experienced flood or storm damage in their neighbourhood. Other studies have found that the risk of mental health difficulties is also elevated in this wider group [44,54], a heightened risk that may moderate the associations we found between storm and flood damage to the home and poorer mental health.

We note some caveats about our study. England’s floods of winter 2013–2014 had a distinctive regional and meteorological profile. Flooding was concentrated in Southern England, where heavy rainfall resulted in fluvial flooding. In December 2013, rainfall was 154% above the national average for that month and January 2014 was the wettest January on record for Southern England. Soils were quickly saturated and rain ran off the land into rivers that subsequently flooded [55]. Because the dataset released for our study included only limited area-level data, we could not take account of location in our study. It, therefore, needs to be complemented by national studies of mental health where flood damage relates to different forms of flooding (e.g., tidal flooding) and/or affects different areas, including England’s less wealthy regions and coastal communities [48,49,50].

The APMS is cross-sectional; it, therefore, has no prospective measures of mental health. In consequence, we could not control for prior mental health, and it was not possible to derive causal inference. However, because storms and floods are infrequent, unpredictable and localized events, prospective studies are the exception and, like the APMS, most studies rely on post-exposure measures of mental health [6]. Our study validates previous work on the mental health impacts of a home being damaged by extreme weather, and provides new evidence on the broader associations of extreme weather damage (beyond flooding) with depression and anxiety disorders. Associations between such exposures and mental health may operate through a range of pathways, including material and financial losses, loss of the psychological security that people derive from their home and locality and disruption of health and social services [37,38,39,40,41,42,44,56,57,58,59].

Finally, while large, the sample was too small to examine some variables robustly (for example, ethnic group), to estimate risks for particular CMDs (for example, depression) or to investigate those reporting suicide attempts.

Studies of the mental health of individuals exposed to flooding often rely on area-level measures, for example, patient lists from GP practices and local authority lists of residential addresses in flood-affected areas [54,60]. However, flooding can result in population displacement [6,54,60], with a consequent under-reporting of adverse health events and increased healthcare use in those moving out of their home [61]. Based on individual-level data, the APMS included those displaced from their homes. We found little evidence of population displacement following weather-related damage to the home (0.1%); this suggests that evidence on mental health impacts based on surveys of properties may miss only a small proportion of the flood-exposed population (albeit those most adversely affected).

## 5. Conclusions

Using a survey of adults in England, we were able to compare the mental health of individuals with and without a recent experience of storm- or flood-related damage to their home in population-wide analyses that took account of a wide range of socio-demographic factors. We found that living in a storm- or flood-related damaged home operated as an additional and independent risk factor for CMD. 

With climate change predicted to increase the frequency and intensity of storms and floods in the U.K., the inclusion of storm- and flood-exposure measures in UK health surveys, both cross-sectional and prospective, should be a priority. Evidence on the short-term and longer-term mental health of affected populations is required to inform disaster preparedness and response planning and to enable support to be targeted at those at heightened risk.

## Figures and Tables

**Table 1:** Prevalence of mental disorders and suicidal thoughts and attempts in total population and by storm- or flood-related damage to the home in previous 6 months.

	Total Population	Storm/Flood Damaged Home in Previous 6 Months				Significance of Association with Storm/Flood Damage *
	% (*n* = 7525)	Yes % (*n* = 354)	95% CI	No % (*n* = 7171)	95% CI	*p* Value
Any common mental disorder (CMD)	17.0 (1329)	23.1 (89)	18.5–28.4	16.7 (1240)	15.7–17.8	0.005
Posttraumatic stress disorder (PTSD) screen positive	4.4 (311)	6.6 (25)	4.3–10.1	4.3 (286)	3.7–5.0	0.062
Suicidal ideation & attempts						
Suicidal thoughts ever	20.5 (1602)	29.5 (114)	24.4–35.2	20.1 (1488)	19.0–21.2	0.001
Suicidal thoughts in the past year	5.0 (390)	8.8 (29)	5.6–13.4	4.8 (361)	4.3–5.4	0.010
Suicide attempt ever	6.7 (549)	11.3 (41)	8.1–15.8	6.5 (508)	5.9–7.2	0.003

* Significance testing was conducted by running a binary logistic regression to test for association between experience of storm- or flood-related damage and each mental health outcome without adjustment for other factors.

**Table 2:** Multiple logistic regression analysis of socioeconomic predictors of storm- or flood-related damage to home.

Factors		%	Unadjusted Odds Ratios				Model 1 ^a^				Model 2 ^b^			
		Storm/Flood Damage	Odds Ratio (OR)	CI Lower	CI Upper	*p* Value	ORs	CI Lower	CI Upper	*p* Value	ORs	CI Lower	CI Upper	*p* Value
Sex	Male (ref)	5.3	1				1				1			
	Female	3.8	0.70	0.54	0.91	0.007	0.74	0.57	0.96	0.021	0.73	0.56	0.94	0.023
Age group	16–24 (ref)	2.9	1			0.582 ^c^	1			0.664 ^c^	1			0.547 ^c^
	25–34	4.6	1.61	0.86	3.02	0.133	1.27	0.67	2.43	0.461	1.32	0.69	2.53	0.393
	35–44	5.4	1.89	1.06	3.37	0.031	1.34	0.76	2.36	0.312	1.39	0.79	2.44	0.259
	45–54	5.3	1.85	1.06	3.25	0.032	1.24	0.71	2.18	0.443	1.28	0.73	2.24	0.395
	55–64	5.2	1.82	1.00	3.31	0.05	1.32	0.72	2.42	0.363	1.27	0.70	2.30	0.421
	65–74	4.3	1.49	0.84	2.62	0.172	1.22	0.64	2.33	0.542	1.05	0.60	1.84	0.853
	75+	3.5	1.22	0.67	2.24	0.517	1.15	0.56	2.35	0.710	0.94	0.51	1.72	0.830
Housing tenure	Owner occ. (ref)	5.4	1			<0.001 ^c^	1			0.002 ^c^	1			0.002 ^c^
	Social renter	3.2	0.58	0.01	0.38	0.010	0.66	0.41	1.05	0.078	0.67	0.42	1.06	0.086
	Private renter	3.0	0.55	0.00	0.38	0.001	0.57	0.38	0.83	0.004	0.57	0.39	0.84	0.005
Equivalised household income quintiles	Highest (ref)	7.2	1			<0.001 ^c^	1			0.002 ^c^	1			<0.001 ^c^
	2	5.2	0.72	0.48	1.07	0.100	0.75	0.50	1.12	0.162	0.75	0.50	1.13	0.170
	3	4.6	0.62	0.40	0.96	0.032	0.72	0.45	1.14	0.158	0.73	0.46	1.15	0.173
	4	5.3	0.73	0.50	1.05	0.085	0.91	0.60	1.39	0.672	0.91	0.62	1.34	0.638
	Lowest	3.3	0.45	0.30	0.67	<0.001	0.61	0.37	1.02	0.06	0.60	0.38	0.96	0.032
	Unknown	2.9	0.38	0.25	0.59	<0.001	0.50	0.31	0.79	0.003	0.50	0.32	0.78	0.002
Employment status	Employed (ref)	5.2	1			0.001 ^c^	1			0.089 ^c^				
	Unemployed	4.2	0.80	0.38	1.71	0.570	1.12	0.50	2.50	0.777				
	Inactive	3.5	0.65	0.50	0.85	0.001	0.77	0.53	1.12	0.173				
Index of Multiple Deprivation (IMD)	Highest (least deprived) (ref)	4.4	1			0.705 ^c^	1			0.251 ^c^				
	2	4.8	1.09	0.75	1.59	0.648	0.81	0.81	1.74	0.382				
	3	5.2	1.19	0.82	1.72	0.356	0.93	0.93	1.97	0.113				
	4	3.9	0.88	0.60	1.30	0.529	0.73	0.73	1.61	0.696				
	Lowest (most deprived)	4.5	1.01	0.67	1.54	0.950	0.83	0.83	2.13	0.236				
Population density	Urban	4.4	1			0.370 ^c^								
	Suburban/small town	5.0	1.13	0.77	1.66	0.523								
	Rural	5.1	1.17	0.78	1.74	0.443								

^a^ Model 1 includes sex and age group, and socioeconomic factors (tenure, equivalised household income, and employment status) that significantly predicted exposure to storm- or flood-related damage in bivariate analysis, plus area-level deprivation (IMD). ^b^ Model 2 includes factors significant in Model 1. ^c^ *p*-values are for the variable as a whole. The mean variance inflation factor (VIF) for Model 1 was 1.27, with the VIFs for each individual variable being below 1.6, indicating no concerns with multicollinearity.

**Table 3:** Multiple logistic regression analysis of predictors of common mental disorder.

Factors		%	Unadjusted				Model 1 ^a^				Model 2 ^b^			
		CMD	Odds Ratios	CI Lower	CI Upper	*p* Value	Odds Ratios	CI Lower	CI Upper	*p* Value	Odds Ratios	CI Lower	CI Upper	*p* Value
Storm/flood damage to home	No (ref)	16.7	1				1				1			
	Yes	23.1	1.50	1.13	1.99	0.005	1.81	1.34	2.44	<0.001	1.50	1.08	2.07	0.014
Sex	Male (ref)	13.1	1				1				1			
	Female	20.6	1.72	1.48	2	<0.001	1.67	1.43	1.95	<0.001	2.05	1.71	2.46	<0.001
Age group	16–24 (ref)	18.8	1			<0.001 ^c^	1			<0.001 ^c^	1			<0.001 ^c^
	25–34	19.1	1.01	0.77	1.33	0.944	1.19	0.88	1.60	0.252	0.92	0.67	1.25	0.584
	35–44	19.3	1.03	0.79	1.34	0.835	1.30	0.97	1.76	0.082	0.89	0.64	1.25	0.505
	45–54	18.9	1.01	0.79	1.3	0.932	1.38	1.03	1.85	0.031	0.70	0.50	0.98	0.040
	55–64	17.9	0.94	0.72	1.22	0.638	1.09	0.81	1.47	0.577	0.51	0.36	0.71	<0.001
	65–74	11.4	0.56	0.41	0.76	<0.001	0.50	0.36	0.70	<0.001	0.28	0.18	0.41	<0.001
	75+	8.8	0.41	0.3	0.56	<0.001	0.32	0.23	0.45	<0.001	0.14	0.09	0.21	<0.001
Housing tenure	Owner occ. (ref)	13.3	1			<0.001 ^c^	1			0.003 ^c^	1			0.933 ^c^
	Social renter	28.7	2.62	2.21	3.11	<0.001	1.71	1.41	2.07	<0.001	1.04	0.83	1.30	0.718
	Private renter	19.4	1.58	1.33	1.88	<0.001	1.28	1.05	1.57	0.014	1.04	0.83	1.31	0.708
Employment status	Employed (ref)	14.6	1			<0.001 ^c^	1			<0.001 ^c^	1			0.152 ^c^
	Unemployed	25.1	1.96	1.37	2.81	<0.001	1.46	1.00	2.14	0.048	1.17	0.75	1.84	0.492
	Inactive	20.1	1.48	1.28	1.7	<0.001	1.96	1.63	2.36	<0.001	1.31	1.06	1.61	0.012
Equivalised household income quintiles	Highest (ref)	11.9				<0.001 ^c^	1			0.112 ^c^	1			0.583 ^c^
	2	12	1.01	0.76	1.34	0.945	1.01	0.76	1.35	0.947	0.91	0.67	1.24	0.553
	3	16.2	1.43	1.1	1.85	0.008	1.25	0.95	1.65	0.116	1.07	0.79	1.44	0.667
	4	17.1	1.53	1.17	1.98	0.002	1.12	0.84	1.49	0.454	0.85	0.62	1.16	0.303
	Lowest	26.8	2.70	2.11	3.45	<0.001	1.47	1.11	1.95	0.008	1.05	0.76	1.44	0.787
	Unknown	17.5	1.59	1.23	2.06	<0.001	1.24	0.93	1.65	0.142	0.97	0.72	1.30	0.829
Index of Multiple Deprivation (IMD)	Highest (least deprived) (ref)	10.8	1			<0.001 ^c^	1			<0.001 ^c^	1			0.002 ^c^
	2	13.7	1.32	1.03	1.69	0.028	1.25	0.97	1.60	0.082	1.20	0.92	1.58	0.177
	3	16.0	1.58	1.26	1.98	<0.001	1.41	1.11	1.78	0.005	1.22	0.94	1.58	0.126
	4	20.4	2.13	1.66	2.74	<0.001	1.70	1.29	2.23	<0.001	1.44	1.08	1.93	0.013
	Lowest (most deprived)	23.9	2.62	2.06	3.34	<0.001	1.75	1.35	2.26	<0.001	1.42	1.08	1.88	0.013
Arrears	Not in arrears (ref)	14.9	1								1			<0.001
	Arrears	41.3	4.02	3.27	4.94	<0.001					2.09	1.60	2.74	<0.001
General health	Excellent (ref)	6.0	1			<0.001 ^c^					1			<0.001 ^c^
	Very good	10.8	1.87	1.39	2.52	<0.001					2.06	1.52	2.80	0.177
	Good	18.6	3.54	2.64	4.75	<0.001					4.09	3.01	5.55	0.126
	Fair	31.1	6.98	5.22	9.34	<0.001					10.17	7.34	14.10	0.013
	Poor	55.0	18.97	13.95	25.79	<0.001					28.94	20.04	41.81	0.013
Alcohol use	Low/no risk (ref)	15.9	1			<0.001 ^c^					1			<0.001 ^c^
	Hazardous	16.9	1.06	0.87	1.30	0.546					1.30	1.03	1.63	0.024
	Harmful	41.6	3.69	2.67	5.10	<0.001					2.95	2.00	4.36	<0.001

^a^ Model 1 includes storm/flood damage, sex and age group, socioeconomic factors (tenure, employment status, equivalised household income) and area-level deprivation (IMD quintiles) that are significant predictors of both CMD and flood damage in bivariate analysis. ^b^ Model 2 includes the factors in Model 1, and further controls for health- and stress-related context by adjusting for being in debt arrears, general health and alcohol use. ^c^ *p*-value are for the variable as a whole. The mean VIF for Model 2 was 1.22, and the VIF for individual variables were all less than 1.7.

## Supplementary Materials

The following are available online at https://www.mdpi.com/1660-4601/16/18/3256/s1, Table S1: Flood damage to home in the past six months, by sex; Table S2: Demographic factors by experience of flood damage to home in past six months; Table S3: Household characteristics of people experiencing flood damage to their home in past six months; Table S4: Income and employment status by experience of flood damage to home in past six months; Table S5: Financial strain and debt indicators by experience of flood damage to their home in past six months; Table S6: Current property and area characteristics by experience of flood damage to home in past six months; Table S7: Common mental disorders and severity of CMD symptoms by experience of flood damage to home in past six months; Table S8:Other mental disorders by experience of flood damage to home in past six months; Table S9: Signs of substance dependence by experience of flood damage to home in past six months; Table S10: Suicidal thoughts, attempt and self-harm by experience of flood damage to home in past six months; Table S11: Mental health treatment and service use by experience of flood damage to home in past six months; Table S12: General health by experience of flood damage to home in past six months.
