# Supplementary material for: Flood- and Weather-Damaged Homes and Mental Health: An Analysis Using England’s Mental Health Survey
Source: Int J Environ Res Public Health. 2019 Sep 5;16(18):3256. doi: 10.3390/ijerph16183256 (PMC6765946; doi:10.3390/ijerph16183256)
Supplement: Supplementary file 1 [file ijerph-16-03256-s001.pdf]

SUPPLEMENTARY MATERIALS

Table S1. **Storm/flood damage to home in the past six months, by sex.**

	Sex		
	**Men**	**Women**	**Total Population**
Experienced in last six months…	%	%	%
Any flood damage	5.3	3.8	4.5
'Severe' flood damage [a]	0.05	0.10	0.08
Bases	3053	4472	7525

Statistical significance of association between any flood damage and sex: $p = 0.007$. [a] *Severe* flood damage was defined as having to leave the home for at least three days or were unable to leave the home for at least three days. Only seven participants reported this.

Table S2. Demographic factors by experience of storm/flood damage to home in past six months.

	Storm/flood damage to home in past six months		
	**Damaged**	**Not**	**Total Population**
Age group	%	%	%
16–34	26	31	31
35–54	39	33	34
55–74	27	26	26
75+	8	10	10
All	100	100	100
Ethnic group [a]			
White British	85	80	81
White other	4	7	7
Black	1	3	3
Asian	7	7	7
Mixed/other	3	3	3
All	100	100	100
Highest ed. qualification			
Degree	31	25	25
Teaching, HND, nursing	14	8	8
A Level	14	19	18
GCSE or equivalent	26	25	25
Foreign/other	2	3	3
No qualifications	13	21	20
All	100	100	100
Bases	354	7171	7525

Statistical significance: age group $p = 0.129$; ethnic group $p = 0.125$; highest educational qualifications $p = 0.028$. [a] The APMS sample is too small to examine variation by ethnic group robustly.

Table S3. Household characteristics of people experiencing storm/flood damage to their home in past six months.

	Storm/flood damage to home in past six months		
	**Damaged**	**Not**	**Total Population**
Housing tenure	%	%	%
Owner occupier	76	63	64
Private rented	11	16	16
Social rented	13	20	20
All	100	100	100
Household type [a]			
1 adult 16–59	11	7	7
2 adults 16–59	19	20	20
Small family	21	16	16
Large family	3	6	6
Several adults	24	26	26
2 adults 60+	15	17	17
1 adult 60+	7	8	8
All	100	100	100
Lone adult with child/ren household	3	2	2
Bases	354	7141	7495

Statistical significance: Tenure $p < 0.001$; Household type $p = 0.002$; Lone adult with child $p = 0.707$. [a] Only the small or large family households contain children. Small family units include 1 or 2 adults and 1 or 2 children. Large family units include at least 2 adults and at least 3 children. '2 adults 60+' includes at least one adult 60+.

Table S4. Income and employment status by experience of storm/flood damage to home in past six months.

	Storm/flood damage to home in past six months		
	**Damaged**	**Not**	**Total Population**
Equivalised household income [a]	%	%	%
Highest quintile	29	20	21
2	20	19	19
3	18	21	21
4	21	20	20
Lowest quintile	13	20	19
All	100	100	100
Bases (note higher levels of missing data)	303	5567	5870
Employment status [b]			
In employment	69	59	60
Unemployed	3	3	3
Economically inactive	28	37	37
All	100	100	100
Bases	354	7171	7525

Statistical significance: Income quintiles $p = 0.002$; Employment status $p = 0.002$. [a] Equivalised household income refers to the household income from all sources, and adjusted for the number, ages and relationships of the people living in the household. Income information is missing for some due to some participants not knowing the total household income and also due to sensitivity about disclosing income. [b] 'Economically inactive' includes people who are students, retired, and those not employed or looking for work because of looking after family.

Table S5. Financial strain and debt indicators by experience of storm/flood damage to their home past six months.

	Storm/flood damage to home in past Six Months		
	**Damaged**	**Not**	**Total population**
	%	%	%
Major financial crisis [a]	17	11	11
Debt arrears [b]	10	7	7
Ever been homeless	5	4	4
Bases	354	7166	7520

Statistical significance: Financial crisis p = 0.002; Debt arrears p = 0.076; Homelessness p = 0.413. [a] Defined as equivalent of loss of three months' income. [b] Debt arrears involved being 'seriously behind' in paying for any of a series of types of debt.

Table S6. Current property and area characteristics by experience of storm/flood damage to home in past six months.

	Storm/flood damage to home in past six months		
	**Damaged**	**Not**	**Total Population**
Index of Multiple Deprivation (IMD)	%	%	%
Highest quintile (most affluent)	19	20	20
2	21	20	20
3	23	20	20
4	17	20	20
Lowest quintile (most deprived)	20	20	20
All	100	100	100
Population density			
Urban	80	82	82
Suburban/small town	10	9	9
Rural	10	9	9
All	100	100	100
Presence of mould in the household [a]	41	20	21
Unable to keep home warm enough in winter [a]	10	7	7
Bases	354	7165	7519

Statistical significance: IMD p = 0.777; Rural p = 0.368; Mould p < 0.001; Warm home p = 0.052. [a] These indicators are sometimes used as proxies for identifying a household that is possibly fuel poor. These relate to the participants' current property, which may not be the same property they were living in when flooded.

Table S7. Common mental disorders (CMD) and severity of CMD symptoms by experience of storm/flood damage to home in past six months.

	Storm/flood damage to home in past six months		
	**Damaged**	**Not**	**Total Population**
	%	%	%
Any common mental disorder (CMD) [a]	23	17	17
Depression	7	3	3
CMD not otherwise specified [b]	11	8	8
Generalised anxiety disorder (GAD)	6	6	6
Obsessive compulsive disorder (OCD)	2	1	1
Panic disorder	0	1	1
Phobias	4	2	2
Severity of CMD symptoms (CIS-R score)			
0–5	59	69	69
6–11	19	16	16
12–17	11	7	8
18 or more	11	8	8
All	100	100	100
Bases	354	7171	7525

Statistical significance: CMD $p = 0.005$; Depression $p < 0.001$; CMD NOS $p = 0.055$; GAD $p = 0.831$; OCD $p = 0.606$; Panic $p = 0.268$; Phobias $p = 0.089$; CIS-R four $p < 0.001$. [a] CMDs consist of depression, phobias, GAD, OCD, panic disorder and CMD NOS (not otherwise specified). It was possible to have more than one type of CMD identified. They cause appreciable emotional distress and interfere with daily function, but do not usually affect insight or cognition. In APMS, CMDs were assessed using the revised Clinical Interview Schedule (CIS-R), which covers non-psychotic symptoms in the past week. Responses were used to generate an overall score and to diagnose six types of CMD (Lewis et al. 1992). [b] The 'CMD not otherwise specified' category is mutually exclusive of the other types of CMD, and generally less severe. [c] A total CIS-R score was also produced, which is an indication of the overall severity of symptoms. CIS-R score of 12 or more is the threshold applied to indicate that a level of CMD symptoms is present such that primary care recognition is warranted. 'Presence of CMD symptoms' includes all participants with a CIS-R score of 12 or more (including those with a score of 18 and above). CIS-R score of 18 or more denotes more severe or pervasive symptoms of a level very likely to warrant intervention such as medication or psychological therapy. 'Severe CMD symptoms' is used to indicate those with a CIS-R score of 18 or more.

Table S8. Other mental disorders by experience of storm/flood damage to home in past six months.

	Storm/flood damage to home in past six months		
	Damaged Home	No Damage	Total Population
	%	%	%
Posttraumatic stress disorder (PTSD) screen positive [a]	7	4	4
Attention-deficit hyperactivity disorder (ADHD) screen positive [b]	11	10	10
Bipolar disorder screen positive [c]	2	2	2
Psychotic disorder [d]	1	0	0
Personality disorder screen positive [e]	19	13	14
Bases	354	7171	7525

Statistical significance: PTSD $p = 0.062$; ADHD $p = 0.415$; Bipolar disorder $p = 0.663$; psychotic disorder p=0.084; any personality disorder p=0.013. [a] PTSD is a disabling condition characterised by flashbacks and nightmares, avoidance and numbing, and hyper-vigilance. It is different from other psychiatric disorders in that diagnosis links symptoms to an external, traumatic event. A traumatic event is where an individual experiences, witnesses, or is confronted with life endangerment, death or serious injury or threat to self or close others. Traumatic events are distinct from and more severe than generally stressful life events. Screening positive on the PTSD Checklist – civilian (PCL-C), administered by self-completion, indicated presence of trauma related symptoms in the past week and that clinical assessment for PTSD was warranted (Fear et al., 2016). [b] ADHD is a developmental disorder consisting of core dimensions of inattention, hyperactivity and impulsiveness. Characteristic symptoms and behaviours include excessive problems with organisation, difficulties with activities requiring cognitive involvement, restlessness and impulsiveness to an extent that causes significant distress or interferes with everyday functioning. A score of four or more on the Adult Self-Report Scale-v1.1 (ASRS) was considered to be a positive screen indicating that a clinical assessment for ADHD may be warranted (Brugha et al., 2016). [c] Bipolar disorder, previously known as manic depression, is a common, lifelong, mental health condition characterised by recurring episodes of depression and mania. It is associated with significant impairment. The 15-item Mood Disorder Questionnaire was added to the 2014 APMS. A positive screen required endorsement of at least 7 lifetime manic/hypomanic symptoms, as well as several co-occurring symptoms, together with moderate or serious functional impairment. A positive screen indicated the likely presence of bipolar disorder and that fuller assessment would be warranted (Marwaha et al., 2016). [d] Psychotic disorders produce disturbances in thinking and perception severe enough to distort perception of reality. APMS participants were diagnosed with 'probable psychosis' if they completed a second phase SCAN (Schedule for Clinical Assessment in Neuropsychiatry) interview and it was positive or where no SCAN was conducted if two or more psychosis screening criteria were endorsed in the phase one interview (Bebbington et al., 2016).[e] Personality disorders are longstanding, ingrained distortions of personality that interfere with the ability to make and sustain relationships. A general personality disorder screen (the SAPAS) was added to APMS 2014 to screen adults of all ages for 'any personality disorder' (PD) (Moran et al., 2016).

Table S9. Signs of substance dependence by experience of storm/flood damage to home in past six months.

	Storm/flood damage to home in past six months		
	**Damaged Home**	**No Damage**	**Total Population**
	%	%	%
Alcohol use [a]			
Low risk (AUDIT 0–7)	72	81	80
Hazardous drinking (AUDIT 8–15)	22	16	17
Harmful/mild dependence (AUDIT 16–19)	4	2	2
Probable dependence (AUDIT 20 +)	2	1	1
All	100	100	100
Drug dependence [b]	7	3	3
Smoking status			
Never smoked	31	40	39
Ex-smoker	51	41	41
Smoker – less than 15 per day	12	13	13
Smoker – 15 or more a day	6	6	6
All	100	100	100
Bases	354	7171	7525

Statistical significance: AUDIT p = 0.002; Drug dependence p = 0.016; Smoking status p = 0.005. [a] Harmful drinking was assessed using the Alcohol Use Disorders Identification Test (AUDIT) (Saunders et al.,, 1993). The AUDIT takes the year before the interview as a reference period, consists of 10 items and covers the following areas: alcohol consumption (frequency of drinking, typical quantity, frequency of heavy drinking); alcohol-related harm (feeling of guilt or remorse after drinking, blackouts, alcohol-related injury, other concern about alcohol consumption); symptoms of alcohol dependence (impaired control over drinking, increased salience of drinking, morning drinking). Answers to all questions are scored from zero to four, and summed to give a total score ranging from 0 to 40. Harmful drinking and/or mild dependence was indicated by a score from 16 to 19; probable dependence with a score of 20 or more (Drummond et al., 2016). [b] Use of a drug and the presence of one of five symptoms of dependence in the past year were used to indicate signs of possible drug dependence, a lower threshold than recommended elsewhere. For each of eight drug types (cannabis, amphetamines, crack, cocaine, ecstasy, tranquillisers, opiates and volatile substances), reported use in the past year was followed by five questions based on the Diagnostic Interview Schedule. These questions asked about the past month and year, and covered: daily use for 2 weeks or more; a sense of need or dependence; an inability to abstain; increased tolerance; and withdrawal symptoms. A positive response to any of the items in the past year was used to indicate drug dependence (Roberts et al., 2016).

Table S10. Suicidal thoughts, attempt and self-harm by experience of storm/flood damage to home in past six months.

	Storm/flood damage to home in past six months		
	Damaged Home	No Damage	Total Population
	%	%	%
Suicidal thoughts ever [a]	30	20	21
Suicidal thoughts in the past year [a]	9	5	5
Self-harm ever [a]	11	6	7
Suicide attempt ever [a]	8	7	7
Bases	354	7171	7525

Statistical significance: Suicidal thoughts ever $p = 0.001$; Suicidal thoughts past year $p = 0.010$; Suicide attempt ever $p = 0.003$; Self-harm ever $p = 0.481$. [a] Suicidal thoughts, non-fatal suicide attempts and self-harm (without suicidal intent) are associated with high levels of distress, both for the people engaging in them and for those around them. Respondents were asked about these in both the face to face and self-completion sections of the interview, these variables were derived from both (McManus et al., 2019).

Table S11. Mental health treatment and service use by experience of storm/flood damage to home in past six months.

	Storm/flood damage to home in past six months		
	Damaged Home	No Damage	Total Population
Current use of mental health treatment [a]	%	%	%
No treatment	83	87	87
Medication only	11	10	10
Counselling only	3	1	2
Both medication and counselling	3	1	1
All	100	100	100
Bases	354	7160	7514

Statistical significance: $p = 0.059$. [a] Treatment consisted of mental health medication and/or psychological counselling at the time of the interview (Lubian et al., 2016).

Table S12. General health by experience of storm/flood damage to home in past six months.

	Storm/flood damage to home in past six months		
	Damaged Home	No Damage	Total Population
Self-reported general health	%	%	%
Excellent	17	21	21
Very good	31	35	35
Good	31	26	26
Fair	14	13	13
Poor	6	6	6
All	100	100	100
Bases	354	7165	7519

Statistical significance: Good/fair/poor health: $p = 0.010$

References

Bebbington, P.E., et al., *Chapter 5: Psychotic disorder*, in *Mental Health and Wellbeing in England: Adult Psychiatric Morbidity Survey 2014: a Survey Carried Out for NHS Digital by NatCen Social Research and the Department of Health Sciences, University of Leicester*. 2016, NHS Digital.

Brugha, T., et al., *Chapter 8: Attention-deficit/ hyperactivity disorder*, in *Mental Health and Wellbeing in England: Adult Psychiatric Morbidity Survey 2014: a Survey Carried Out for NHS Digital by NatCen Social Research and the Department of Health Sciences, University of Leicester*. 2016, NHS Digital.

Drummond, C., et al., *Chapter 10: Alcohol dependence*, in *Mental Health and Wellbeing in England: Adult Psychiatric Morbidity Survey 2014: a Survey Carried Out for NHS Digital by NatCen Social Research and the Department of Health Sciences, University of Leicester*. 2016, NHS Digital.

Fear, N.T., et al., *Chapter 4: Post-traumatic stress disorder*, in *Mental Health and Wellbeing in England: Adult Psychiatric Morbidity Survey 2014: a Survey Carried Out for NHS Digital by NatCen Social Research and the Department of Health Sciences, University of Leicester*. 2016, NHS Digital.

Lewis. G., et al. Measuring psychiatric disorder in the community; a standardised assessment for use by lay interviewers. *Psychological Medicine*, 1992; 22: 465–486.

Lubian, K., et al., *Chapter 3: Mental health treatment and services,* in *Mental health and wellbeing in England: Adult Psychiatric Morbidity Survey 2014: a Survey Carried Out for NHS Digital by NatCen Social Research and the Department of Health Sciences, University of Leicester*. 2016, NHS Digital.

Marwaha, S. et al., *Chapter 9: Bipolar disorder*, in *Mental Health and Wellbeing in England: Adult Psychiatric Morbidity Survey 2014: a Survey Carried Out for NHS Digital by NatCen Social Research and the Department of Health Sciences, University of Leicester*. 2016, NHS Digital.

McManus, S., et al., Prevalence of non-suicidal self-harm and service contact in England, 2000–14: repeated cross-sectional surveys of the general population. *Lancet Psychiatry*. 2019: 6, 7, 573-581.

Moran, P., et al., *Chapter 7: Personality disorder*, in *Mental Health and Wellbeing in England: Adult Psychiatric Morbidity Survey 2014: a Survey Carried Out for NHS Digital by NatCen Social Research and the Department of Health Sciences, University of Leicester*. 2016, NHS Digital.

Roberts, C., et al., *Chapter 11: Drug use and dependence*, in *Mental Health and Wellbeing in England: Adult Psychiatric Morbidity Survey 2014: a Survey Carried Out for NHS Digital by NatCen Social Research and the Department of Health Sciences, University of Leicester*. 2016, NHS Digital.

Saunders, J.B., et al., Development of the alcohol use disorders identification test (AUDIT): WHO collaborative project on early detection of persons with harmful alcohol consumption-II. *Addiction*, 1993. **88**(6): p. 791-804.
